# How Long Halt Ye?

**Published:** 1921-01-15

**Authors:** T. Glenton Kerr

**Affiliations:** *Secretary*. The Queen's Hospital for Children, Hackney Road, Bethnal Green, E. 2


*To the Editor of* The Hospital.

### "How Long Halt Ye?"

*To the Editor of* The Hospital.

Sir,—Both the writer of your article "Discussion of a Policy" and Mr. Coles having (if they will forgive me for saying so) somewhat darkened counsel by certain misapprehensions, I must beg some more of your valuable space in order to clear the ground.

1. I did not state that the London weekly wage earners were mainly contributors to the Hospital Saturday Fund, but mentioned that great organisation as the chief among many that are in the field busily occupied in collecting hospital contributions from the working-class population. Those who escape the nets of these fishers are caught by the many other collections frequently made. There can be no doubt of the truth of my statement that the vast majority of weekly wage earners are already contributing liberally to hospitals, and it is a strange thing that this fact should appear to be generally unknown to those who write on the subject of our voluntary system.

2. I did not write to discourage Mr. Coles in his laudable efforts to increase hospital revenue, but to point out what is being already done in the same direction and to put the matter in a true light, so that he and others concerned might not have any unjustifiable expectations as to the possible results of putting his plan into practice in London. Undoubtedly some additional revenue could be raised by endeavours to arrange for a universal voluntary levy, but it is of no use to ignore what is already being done. The best line of advance, in my opinion, would be through the Hospital Saturday Fund and the League of Mercy, which, having been long in the field, possess the necessary machinery and the confidence of the public. There is no reason why there should not be Hospital Saturday Fund collections in every mercantile office in London, as well as in the workshops and warehouses.

These two organisations, together with King Edward's Hospital Fund and the Hospital Sunday Fund, should sweep the whole of London. We should not get more money by holding out more hats—enough are being passed round already—but only by creating the necessary atmosphere.

3. Mr. Coles, in his rejoinder to my letter in your issue of December 18, refers to the statement that "only 10 per cent. of the population contribute to hospitals," as if it were an established fact, and adduces it as evidence against my contention that most of the weekly wage earners are already giving. I challenge the accuracy of this estimate, as I am sure 10 per cent. is below the actual figure, but even if it were correct it would mean that quite 40 per cent. of those who could reasonably be expected to give anything were already helping.

T. Glenton Kerr, *Secretary*.

The Queen's Hospital for Children,

Hackney Road, Bethnal Green, E. 2.

[We regret that we are unable to give further space to correspondence on this subject.—Ed. The Hospital.]

